# Hepatitis B Infection, Viral Load and Resistance in HIV-Infected Patients in Mozambique and Zambia

**DOI:** 10.1371/journal.pone.0152043

**Published:** 2016-03-31

**Authors:** Gilles Wandeler, Kalo Musukuma, Samuel Zürcher, Michael J. Vinikoor, Jara Llenas-García, Mussa M. Aly, Lloyd Mulenga, Benjamin H. Chi, Jochen Ehmer, Michael A. Hobbins, Carolyn Bolton-Moore, Christopher J. Hoffmann, Matthias Egger, IeDEA-Southern Africa

**Affiliations:** 1 Department of Infectious Diseases, Bern University Hospital, University of Bern, Bern, Switzerland; 2 Institute of Social and Preventive Medicine, University of Bern, Bern, Switzerland; 3 Department of Infectious diseases, University of Dakar, Dakar, Senegal; 4 Centre for Infectious Disease Research in Zambia, Lusaka, Zambia; 5 Institute for Infectious Diseases, University of Bern, Bern, Switzerland; 6 Department of Medicine at University of Alabama, Birmingham, United States of America; 7 SolidarMed, Ancuabe, Mozambique; 8 Nucleo do investigacão Operational de Pemba, Pemba, Mozambique; 9 Department of Obstetrics and Gynecology, University of North Carolina, Chapel Hill, United States of America; 10 SolidarMed, Lucerne, Switzerland; 11 Johns Hopkins University School of Medicine, Baltimore, United States of America; 12 Centre for Infectious Disease Epidemiology and Research, University of Cape Town, Cape Town, South Africa; CRCL-INSERM, FRANCE

## Abstract

**Background:**

Few data on the virological determinants of hepatitis B virus (HBV) infection are available from southern Africa.

**Methods:**

We enrolled consecutive HIV-infected adult patients initiating antiretroviral therapy (ART) at two urban clinics in Zambia and four rural clinics in Northern Mozambique between May 2013 and August 2014. HBsAg screening was performed using the Determine^®^ rapid test. Quantitative real-time PCR and HBV sequencing were performed in HBsAg-positive patients. Risk factors for HBV infection were evaluated using Chi-square and Mann-Whitney tests and associations between baseline characteristics and high level HBV replication explored in multivariable logistic regression.

**Results:**

Seventy-eight of 1,032 participants in Mozambique (7.6%, 95% confidence interval [CI]: 6.1–9.3) and 90 of 797 in Zambia (11.3%, 95% CI: 9.3–13.4) were HBsAg-positive. HBsAg-positive individuals were less likely to be female compared to HBsAg-negative ones (52.3% vs. 66.1%, p<0.001). Among 156 (92.9%) HBsAg-positive patients with an available measurement, median HBV viral load was 13,645 IU/mL (interquartile range: 192–8,617,488 IU/mL) and 77 (49.4%) had high values (>20,000 UI/mL). HBsAg-positive individuals had higher levels of ALT and AST compared to HBsAg-negative ones (both p<0.001). In multivariable analyses, male sex (adjusted odds ratio: 2.59, 95% CI: 1.22–5.53) and CD4 cell count below 200/μl (2.58, 1.20–5.54) were associated with high HBV DNA. HBV genotypes A1 (58.8%) and E (38.2%) were most prevalent. Four patients had probable resistance to lamivudine and/or entecavir.

**Conclusion:**

One half of HBsAg-positive patients demonstrated high HBV viremia, supporting the early initiation of tenofovir-containing ART in HIV/HBV-coinfected adults.

## Introduction

Chronic hepatitis B virus (HBV) infection is present in approximately 3 million HIV-infected persons worldwide [1]. HIV/HBV-coinfected individuals are at higher risk of death and are more likely to develop hepatic complications and to have an impaired immunological recovery during antiretroviral therapy (ART), compared to HIV-monoinfected patients [2–4]. Although approximately 10% of HIV-infected patients are estimated to be HBsAg-positive in sub-Saharan Africa (SSA) [5], only few studies have assessed the level of HBV replication and drug resistance in this part of the world.

HBV viral load is a strong predictor of the risk of developing hepatocellular carcinoma and plays an important role in treatment success. Time to HBV suppression under tenofovir (TDF) is delayed with increasing baseline HBV DNA and the risk of persistent viral replication after two years of therapy is highest with an elevated pre-ART HBV viral load [6, 7]. In one of few published reports on HBV outcomes following TDF-containing ART in SSA, pre-therapy HBV-DNA was the only factor independently associated with sub-optimal HBV response [8]. In that study, which included 55 HIV/HBV-coinfected individuals from Zambia, HBV genotype A1 predominated (76%) and only 2 patients had drug-resistance mutations in the HBV polymerase. Although several small studies from other countries in southern Africa have reported HBV sequencing data among ART-naïve HIV/HBV-coinfected populations [9–11], no large study has been conducted in Mozambique and Zambia to evaluate the HBV genotype distribution and drug resistance mutations.

We assessed the prevalence of HBV infection in HIV-infected individuals from an international collaboration including ART programs in Mozambique and Zambia, and performed a detailed analysis of demographic, clinical and virological characteristics associated with high pre-ART HBV DNA. We also studied the distribution of HBV genotypes and the prevalence of pre-ART HBV drug resistance mutations to add to the scarce data from Southern Africa [9, 12].

## Materials and Methods

### Study population

Consecutive HIV-infected adults initiating ART at two urban clinics in Lusaka, Zambia, and four rural clinics in Cabo Delgado, Northern Mozambique were enrolled between May 2013 and August 2014. They were classified as HIV/HBV-coinfected if HBsAg-positive. Routine pre-ART assessment consisted of a physical examination and measurements of CD4 cells, liver transaminases, creatinine and full blood count according to national recommendations. All data were entered into an electronic database, using the protocol of the International epidemiological Databases to Evaluate AIDS in Southern Africa (IeDEA-SA) [13]. Written informed consent was obtained from all patients. Ethics approval was obtained from the Biomedical Research Ethics Committee of the University of Zambia School of Medicine, the Institutional Review Board of University of North Carolina at Chapel Hill, USA, and from the Comité Nacional de Bioética para a Saúde, República de Moçambique.

### Laboratory analyses

HBsAg was assessed using the Determine^®^ (Alere, Yavne, Israel) rapid test on whole blood using finger prick sampling. To determine HBV DNA levels, quantitative real-time polymerase chain reaction (PCR) was performed on plasma samples from Zambian participants and on dried blood spots (DBS) from Mozambican participants using the COBAS Ampliprep/TaqMan System (Roche diagnostics, Indianapolis, USA). We recently showed that the use of DBS to measure HBV DNA level in southern Africa was feasible and reliable [14]. As in our Zambian laboratory DNA measurements from paired plasma samples were in average 1.59 log higher than those from DBS, we converted DNA values from DBS to generate estimated plasma values using a multiplication factor of 39. DNA extraction and amplification procedures used for DBS are explained in detail elsewhere [14]. An in-house protocol was used for HBV sequencing: HBV DNA was extracted using the QIAamp DNA mini kit (Qiagen, Hilden, Germany), a primary PCR covering the codons 18 to 330 of the RT-domain of the Pol gene was conducted using publically available primers [15] and, if necessary, a nested PCR (codons 83–288) was performed using published primers [16]. DNA was purified using QIAquick PCR purification kit (Qiagen, Hilden, Germany). Sanger sequencing was performed using an AB3130 genetic analyzer (Life Technologies, California, USA) and nucleotide sequences analyzed with Sequencher version 5.0 (Gene Codes Corporation, Michigan, USA). Geno2Pheno (www.geno2pheno.org) was used to predict HBV genotypes and drug resistances. HBeAg serology was only available from participants in Zambia. Laboratory analyses were performed at the Centre for Infectious Disease Research in Zambia Central Laboratory and at the Institute for Infectious Diseases, University of Bern.

### Statistical analyses

HBV prevalence estimates were calculated with 95% confidence intervals (CI). Factors associated with HBsAg-positivity were evaluated using Chi-square and Mann-Whitney tests. Associations between demographic, clinical and virological characteristics with the presence of high HBV viral load (≥20,000 IU/mL [17]) were evaluated using the Chi-square test. An AST-to-platelet-ratio-index (APRI) score>1.5 was used to define significant liver fibrosis [18, 19]. Factors associated with high HBV DNA level were further explored in multivariable logistic regression analyses adjusted for sex, age, stage of HIV disease and CD4 cell counts. All statistical analyses were performed using Stata software version 13.1 (College Station, Texas, USA).

## Results

### Demographic and clinical characteristics

Seventy-eight of 1,032 participants in Mozambique (7.6%, 95% confidence interval [CI]: 6.1–9.3) and 90 of 797 in Zambia (11.3%, 95% CI: 9.3–13.4) were HBsAg-positive. HBsAg-positive individuals were less likely to be female compared to those who tested HBsAg-negative (52.3% vs.66.1%, p<0.001) but age, WHO stage, BMI, CD4, haemoglobin and platelets were similar in both groups (Table 1). The results were consistent when comparisons were stratified by country. Median ALT and AST levels were slightly higher in HBsAg-positive patients compared to negative ones.

**Table 1:** Baseline characteristics, by HBsAg-positivity status.

	HBsAg-positive	HBsAg-negative	P-value
	N = 168	N = 1,661	
General characteristics			
Female (%)	88 (52.3)	1,098 (66.1)	<0.001
Median age in years (IQR)	33 (27–39)	32 (26–40)	0.59
WHO stage III/IV (%)	69 (41.3)	625 (37.9)	0.39
Median BMI (IQR)	20 (18–22)	20 (18–23)	0.72
Median first CD4 count in cells/μl (IQR)	229 (116–385)	255 (135–266)	0.71
Median haemoglobin in g/L (IQR)	11.3 (9.4–13.1)	11.1 (9.6–12.7)	0.80
Median platelets x10^9^/L (IQR)	239 (185–301)	244 (189–316)	0.29
Median ALT* in IU/mL (IQR)	24 (15–44)	21 (14–34)	0.10
Median AST* in IU/mL (IQR)	38 (26–60)	32 (25–45)	0.01
HBV characteristics			
Genotype, n = 102		NA	
A1 (%)	60 (58.8)		
E (%)	39 (38.2)		
A1 and E (%)	1 (1.0)		
A2 (%)	2 (2.0)		
Median viral load in IU/mL (IQR)	13645 (192–8617488)	NA	
Viral load>20,000 IU/mL (%)	77 (49.4)	NA	
HBeAg-positivity (%)**	24 (33.3)	NA	

*only available for 66% of the study population

**only available in Zambia (n = 72)

NA: not applicable

### HBV replication at ART initiation

**Fig 1:**
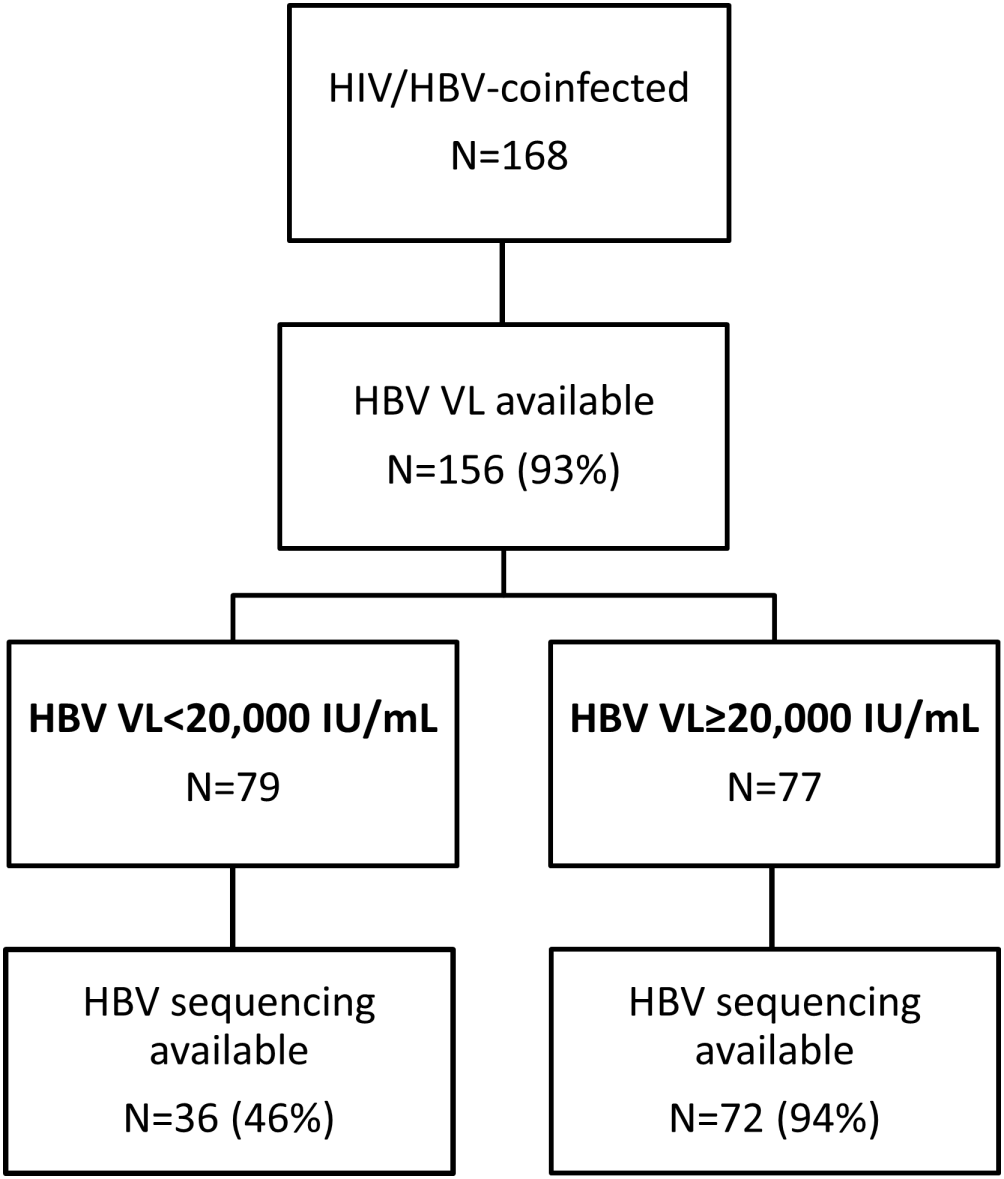
Flow chart of patients included in virological analyses. (HBV: hepatitis B virus; VL: viral load)

**Fig 2:**
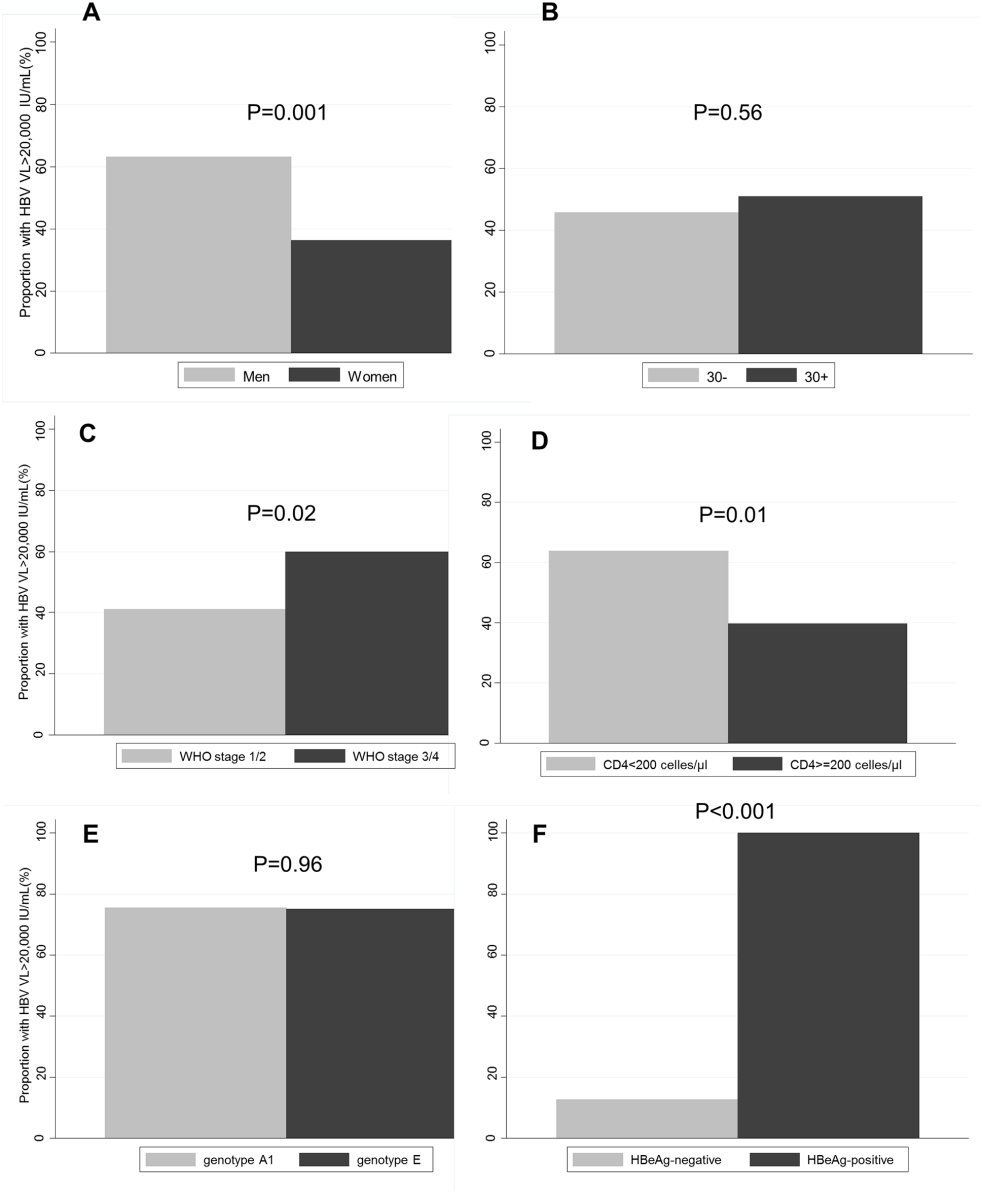
Proportion of patients with high HBV viral load (>20,000 IU/mL), by sex (Panel A), age (B), WHO stage (C), CD4 cell count (D), HBV genotype (E) and HBeAg-positivity (F).

Among HBsAg-positive patients, 156 (92.9%) had an available pre-ART HBV DNA measurement (Fig 1). Median DNA level was 13,645 IU/mL (interquartile range [IQR]: 192–8,617,488). Overall, 26 (16.7%) had an undetectable viral load (<20 IU/mL) and 77 (49.4%) had high values (>20,000 IU/mL). Men were more likely to have high HBV DNA than women (p = 0.01), whereas the proportion of patients with high DNA was highest in patients with advanced WHO stage of HIV disease (p = 0.02) and low CD4 cell counts (p = 0.001, Fig 2). Age and HBV genotype were not associated with the presence of a high HBV DNA level. Of 72 patients with available HBeAg serology, 33.3% had a positive result. All HBeAg+ participants had an HBV DNA>20,000 IU/mL, whereas a high HBV DNA level was only present in 12.8% of HBeAg- individuals (p<0.001, Fig 2). In multivariable analyses, male sex (adjusted odd ratio [aOR]: 2.59, 95% CI: 1.22–5.53) and CD4 cell count below 200/μl (aOR: 2.58, 95% CI: 1.20–5.54) remained associated with having a high pre-ART HBV viral load.

Median transaminase levels were higher in participants with high HBV DNA compared to those with low levels: median ALT: 38 IU/mL (IQR: 19–64) in high HBV DNA vs. 18 (12–24) in low DNA (p<0.001), median AST: 45 IU/mL (32–71) in high HBV DNA and 29 (22–51) in low DNA (p<0.001). Among individuals with high DNA levels, 8 (13.8%) had an APRI score>1.5, whereas this was only the case for 4 (6.7%) patients with low HBV DNA levels (p = 0.20).

### Drug resistance

Among 102 patients with available HBV sequencing results, 58.8% had genotype A1, 38.2% genotype E, one patient had a dual A1/E infection and two had genotype A2 (Table 1). In Mozambique, the prevalence of genotype A1 was higher than in Zambia (72.1% vs. 49.2%). There was no difference in the proportion of HBeAg-positive patients by genotype in Zambia (43.5% in genotype A1 and 60.9% in genotype E, p = 0.24). Three patients showed evidence of lamivudine (3TC)-resistances, three had potential resistance to entecavir (ETV) and five had 3TC or ETV compensatory mutations (Table 2). Three participants had HBV viruses with potentially limited susceptibility to TDF.

**Table 2:** Baseline HBV drug resistance mutations,

Patient	Country	Viral load (IU/ml)	Genotype	Resistance mutations	Drug affected
Drug resistance or limited susceptibility					
1	Mozambique	390	A1	N236T	TDF^l^, ADV^r^
2	Mozambique	1,170	A2	L180M, S202G, M204V	3TC^r^, ETV^r^, LdT^r^
3	Mozambique	2,223	E	N236T	TDF^l^, ADV^r^
4	Mozambique	23,646,012	A1	A194T*	TDF^l^
5	Mozambique	549,900,000	A1	M250I*	ETV^p^
6	Zambia	100,094,677	A1	L180LM, M204MV	3TC^r^, ETV^l^, LdT^r^
7	Zambia	>170,000,000	E	V173L, L180M	3TC^c^^,^^l^, ETV^c^
Compensatory mutations only					
8	Mozambique	21,489	A1	I169L	ETV^c^
9	Mozambique	2,160,561	A1	I169L	ETV^c^
10	Mozambique	43,001,868	A1	M250V	ETV^c^
11	Mozambique	225,233,385	A1	V173L	3TC^c^
12	Zambia	377,982	A1	V173LV	3TC^c^

TDF: tenofovir, ETV: entecavir, 3TC: lamivudine, ADV: adefovir, LdT: telbivudine

^p^ possible resistance,

^l^ limited susceptibility,

^c^ compensatory mutation,

^r^ resistance

*Only according to the Stanford Database for HBV Drug Resistance

## Discussion

In this cohort of HIV-infected individuals starting ART in two distinct settings in southern Africa, HBV prevalence was 8% in rural Mozambique and 11% in urban Zambia. Approximately one half of HBV-coinfected patients had an HBV DNA level≥20,000 IU/mL, with male sex, low CD4 cell counts and HBeAg-positivity being the most important risk factors for having a high viral load. Patients with high HBV DNA levels had higher transaminase levels and were more likely to have significant liver fibrosis thank those with low HBV DNA.

According to a recent meta-analysis, the prevalence of HBsAg-positivity among HIV-infected populations in southern Africa is estimated to reach 5.4% [20]. However, this study considered a large number of reports which were not generalizable to the general population and included only one study from Zambia and none from Mozambique. The higher estimates found in our study were based on the findings of the systematic HBsAg-screening in two large cohorts of general HIV outpatient care. They are comparable to previous findings from an outpatient cohort (9% of HBsAg-positivity in HIV-infected individuals) and inpatient study (10%) in Zambia as well as to the results of a blood donor study in urban Mozambique (10%) [8, 21, 22]. One small study of blood donor candidates from Tete Province in rural Mozambique found an even higher prevalence of HBV-infection among HIV-positive individuals.(14%) [23]. Large prevalence studies are needed from other regions in Mozambique and Zambia to better characterize local epidemiological patterns for HBV infection.

Most studies which assessed HBV replication in patients starting ART in SSA reported high proportions (>50%) of HIV/HBV-coinfected patients with low HBV DNA levels [24–26]. In our study, one half of HIV/HBV-coinfected patients had a viral load≥20,000 IU/mL. We found that all HBeAg-positive Zambian patients had a high DNA levels, whereas the majority of HBeAg-negative individuals had a low DNA levels, a known association also recently reported in Malawi [9]. Thus, in settings with limited access to HBV DNA assays, HBeAg testing could be a valuable tool to identify HBV-infected patients at high risk of liver disease. However, these results need to be confirmed in other clinical settings as other studies from sub-Saharan Africa reported lower correlation between HBeAg-positivity and level of HBV replication.

Like other studies in Southern Africa, we found a high prevalence of HBV genotype A1 [8, 9]. However, over one-third of HBsAg-positive patients were infected with genotype E, which contrasted with the 1% reported in Malawi [9]. Recent studies have shown very low proportions of relevant HBV drug resistance mutations in ART-naïve individuals in Malawi and Zambia [8, 9], whereas 10% of patients had significant drug resistance mutations in an earlier report from South Africa [11]. In our study, 7% (7/102) of patients had potential resistance mutations to 3TC, ETV or TDF, which would most likely not affect treatment outcomes if patients were prescribed TDF. The rtA194T HBV polymerase mutation, recently described in TDF-treated HIV/HBV-coinfected patients in Spain and present in one of our participant from Mozambique was shown to confer no or only partial resistance to TDF in vitro [27–29]. Furthermore, the mutation N236T was associated with reduced activity of TDF in vitro but these data were never confirmed in clinical samples [30]. However, the presence of entecavir-related mutations, present in two patients from Mozambique and one from Zambia, might be important if access to this drug improves in the future, especially for patients with TDF-related nephropathy [31].

Our study contributes detailed virological and clinical data from a large sample of HIV/HBV-coinfected patients starting ART in Mozambique and Zambia, two countries with very limited data available to date. However, our results were limited by missing data on HBeAg in patients from Mozambique and on non-invasive liver fibrosis scores in both countries. Although there were no major differences in clinical characteristics between patients with and without measurements, care must be taken when interpreting data relating to these parameters. Our findings underline the importance of universal HBV testing for HIV-infected populations and early ART including TDF in HIV/HBV-coinfected patients as the level of HBV replication is increased in patients with low cellular immunity and is associated with liver inflammation and fibrosis. With TDF becoming increasingly available for HIV-infected individuals throughout SSA, more data on the main determinants of HBV-infection are needed to inform treatment guidelines.
